# Safety and efficacy of the Seraph® 100 Microbind® Affinity Blood Filter to remove bacteria from the blood stream: results of the first in human study

**DOI:** 10.1186/s13054-022-04044-7

**Published:** 2022-06-17

**Authors:** Gabriele Eden, Julius J. Schmidt, Stefan Büttner, Philipp Kümpers, Carsten Hafer, Alexandros Rovas, Benjamin Florian Koch, Bernhard M. W. Schmidt, Jan T. Kielstein

**Affiliations:** 1 Medical Clinic V Nephrology, Rheumatology, Blood Purification, Academic Teaching Hospital, Braunschweig, Germany; 2 Department of Nephrology and Hypertension, Hannover Medical School, Carl-Neuberg Str. 1, 30625 Hannover, Germany; 3 Medizinische Klinik I - Klinikum Aschaffenburg-Alzenau, Aschaffenburg, Germany; 4 Medical Clinic III, Department of Nephrology, University Hospital Frankfurt, Goethe University, Frankfurt am Main, Germany; 5 Department of Medicine D, Division of General Internal and Emergency Medicine, Nephrology, and Rheumatology, University Hospital Münster, Münster, Germany

**Keywords:** Extracorporeal devices, Bloodstream infections, Haemodialysis, Bacteraemia

## Abstract

**Background:**

Bacterial burden as well as duration of bacteremia influence the outcome of patients with bloodstream infections. Promptly decreasing bacterial load in the blood by using extracorporeal devices in addition to anti-infective therapy has recently been explored. Preclinical studies with the Seraph® 100 Microbind® Affinity Blood Filter (Seraph® 100), which consists of heparin that is covalently bound to polymer beads, have demonstrated an effective binding of bacteria and viruses. Pathogens adhere to the heparin coated polymer beads in the adsorber as they would normally do to heparan sulfate on cell surfaces. Using this biomimetic principle, the Seraph® 100 could help to decrease bacterial burden in vivo.

**Methods:**

This first in human, prospective, multicenter, non-randomized interventional study included patients with blood culture positive bloodstream infection and the need for kidney replacement therapy as an adjunctive treatment for bloodstream infections. We performed a single four-hour hemoperfusion treatment with the Seraph® 100 in conjunction with a dialysis procedure. Post procedure follow up was 14 days.

**Results:**

Fifteen hemodialysis patients (3F/12 M, age 74.0 [68.0–78.5] years, dialysis vintage 28.0 [11.0–45.0] months) were enrolled. Seraph® 100 treatment started 66.4 [45.7–80.6] hours after the initial positive blood culture was drawn. During the treatment with the Seraph® 100 with a median blood flow of 285 [225–300] ml/min no device or treatment related adverse events were reported. Blood pressure and heart rate remained stable while peripheral oxygen saturation improved during the treatment from 98.0 [92.5–98.0] to 99.0 [98.0–99.5] %; *p* = 0.0184. Four patients still had positive blood culture at the start of Seraph® 100 treatment. In one patient blood cultures turned negative during treatment. The time to positivity (TTP) was increased between inflow and outflow blood cultures by 36 [− 7.2 to 96.3] minutes. However, overall TTP increase was not statistical significant.

**Conclusions:**

Seraph® 100 treatment was well tolerated. Adding Seraph® 100 to antibiotics early in the course of bacteremia might result in a faster resolution of bloodstream infections, which has to be evaluated in further studies**.**

*Trail registration*: ClinicalTrials.gov Identifier: NCT02914132, first posted September 26, 2016.

**Supplementary Information:**

The online version contains supplementary material available at 10.1186/s13054-022-04044-7.

## Background

The total bacterial burden and the duration of bacteraemia affects the outcome of patients with blood stream infections. This has been first shown in a preclinical model of pneumonia in which all rabbits receiving small doses of *Streptococcus pneumoniae* survived, while dose escalation of bacterial count increased mortality stepwise up to 100% [1]. In men this correlation between bacterial load and outcome has been shown in *E. coli* and *Staphylococcus aureus* bacteremia where the amount of bacterial DNA in the blood correlated with mortality [2]. The time to positivity (TTP) of blood cultures can be used as an indicator of pathogen load. In 742 adult hospital patients a positive blood culture within 48 to 96 h after initial diagnosis was the strongest predictor of complicated *Staphylococcus aureus* bacteremia [3]. In children with *Staphylococcus aureus* bacteremia a time to positivity of ≤ 17 h correlated with adverse outcomes [4]. The relationship between high bacterial burden and adverse clinical outcome has also been shown for *Neisseria meningitides* [5], *Streptococcus pneumoniae* pneumonia in adults [6] and in children [7]. Aside from surgical intervention, only the timely administration of an empiric, broad-spectrum antibiotic therapy is able to improve outcome in critically ill patient with an infection so far [8]. However, empiric broad spectrum antibiotic treatment increasingly fails due to a rising frequency of resistance among most human pathogens [9], rendering even antibiotics of last resort ineffective [10].

To quickly reduce the blood pathogen load and to be effective in removing even multi-resistant bacteria, extracorporeal methods capturing bacteria are developed by several companies. A fast decrease of blood pathogen content, irrespective of the infectious agent, would not even require the microbiological identification of bacteria and viruses. After failed attempts of such a strategy in the 1980s [11] many different technologies such as filtration [12], use of human opsonin Mannose Binding Lectin [13], magnetic nanoparticle separation [14] bendable polycrystalline nanowires/carbon foam [15] and polyethylene beads with endpoint-attached heparin [16] have been developed. The latter uses covalently end-point attached heparin-coated ultrahigh molecular weight polyethylene (UHMWPE) that mimics heparan sulfate on cell surfaces so that bacteria and viruses bind to it in vitro [17].

The aim of the current first in human study was to investigate safety as well as efficacy of the Seraph® 100 Microbind® Affinity Blood Filter in the setting of blood stream infections in hemodialysis patients.

## Methods

### Patients and study design

This first in human, prospective, multicenter, non-randomized interventional study included patients with bloodstream infections diagnosed with positive blood cultures and the preexisting need for kidney replacement therapy. The study was conducted in four different German hospitals (Academic Teaching Hospital Braunschweig, Hannover Medical School, University Hospital Frankfurt and University Hospital Münster). All patients had an already existing hemodialysis dialysis access at study enrollment, so no dialysis catheters had to be placed. Patients between 18 and 90 years of age were eligible when they showed signs of dialysis catheter related infection (redness, tenderness or purulence at the exit site or a lower time to positivity (TTP) of at least two hours at the catheter than in the peripheral blood culture) or a suspected high bacterial burden (TTP less than 14 h or blood culture positivity in at least two blood cultures). Detailed inclusion and exclusion criteria are shown in Additional file 1: Table S1.

### Medical device and treatment characteristics

The Seraph® 100 Microbind® Affinity Blood Filter (Seraph® 100) was manufactured by ExThera Medical Corporation, Martinez, CA, USA. The Seraph® 100 is a whole blood sorbent hemoperfusion device packed with polymer beads whose surface is modified with covalent end-point attached heparin [17]. This bound heparin mimics cell-surface heparan sulfate in the body, a binding target for pathogens, toxins and Antithrombin III. This gives the Seraph® 100 the ability to remove pathogens while presenting a blood compatible surface to the blood being treated. The Seraph® 100 was placed upstream of the dialyzer on a regular dialysis machine. The treatment was intended to last between three to four hours with an established blood flow of 250–350 mL/min.

### Outcome measures

The *primary endpoint* measure of the study was to demonstrate the safety of the Seraph® 100 in a hemodialysis circuit assessed by rate of adverse events during procedure, clinically significant changes in hematology and chemistry indices and device complications during the procedure and the 14 days thereafter. The *secondary endpoint* measure was the reduction of susceptible or multi-drug resistant (MDR) bacteria in blood passed through the Seraph® 100, indicated by an increase in time to positivity (TTP) of at least 22 min, indicating a pathogen reduction of at least 40% measured as colony forming units (CFU), in those blood cultures with a pre cartridge detectable bacterial count. As reported by Idelevich et al. [18], the relationship between TTP and CFU is inverse and log-linear over the range of 1 to 100 CFU/mL. Therefore a TTP increase of 22 min is estimated to indicate a CFU reduction of about 40%.

### Blood sampling during the treatment

Blood cultures were drawn upstream (inflow) and downstream (outflow) of the Seraph® 100 at 5, 30, 60, 120, 180 and 240 min after treatment initiation. The bacterial burden was assessed by the time to positivity (TTP), defined by the time between collection of the blood culture and the first signal of bacterial growth. Additionally, a panel of routine laboratory test was performed before and after the Seraph® 100 treatment.

### Statistical analysis

To evaluate the safety of the device, which was the primary endpoint of the study, the notified body suggested to include a total of 15 patient in this first in men study. According to a possible 66% pathogen reduction from in-vitro studies, the inclusion of three patients would provide 80% power to confirm the efficacy hypothesis of > 40% pathogen reduction (increase in TTP > 20 min). Categorical variables were summarized using frequencies and percentages of patients in each category. Quantitative variables were expressed as median [Q1–Q3]. Continuous data was compared with the use of Wilcoxon paired signed rank test. For all test used a significance level of *p* < 0.05 was considered significant.

GraphPad Prism 9 (San Diego, CA, USA) and R Statistical Software (version 4.0.4; R Foundation for Statistical Computing, Vienna, Austria) was used for statistical analysis.

## Results

We enrolled fifteen chronic hemodialysis patients (three female and twelve male) with a median age of 74.0 [68.0–78.5] years and a dialysis vintage of 28.0 [11.0–45.0] months. The vascular access comprised of tunneled dialysis catheter (*n* = 9), acute dialysis catheters (*n* = 4) and native AV-fistulas (*n* = 3) as some patients had more than one access. Heparin was used as an anticoagulant in all patients (unfractionated heparin, *n* = 14; low molecular weight heparin, *n* = 1). Patients were treated 66.4 [45.7–80.6] hours after initial positive blood culture. Detailed patient characteristics are given in Table 1.

**Table 1:** Baseline and treatment characteristics of the enrolled patients (data shown as median IQR)

Sex (F/M)	Age (years)	BMI (kg)	Dialysis vintage (months)	Blood flow (ml/min)	Dialysate flow (ml/min)	Ultrafiltration volume (ml)	Time enrolment to treatment (h)	Pathogen
F	72	42.5	86	250	250	0	89.25	*Staph. aureus*
M	74	24.0	39	285	500	1000	138.75	*E. coli*
M	69	24.3	15	81	81	0	45.0	*Staph. aureus*
M	57	33.3	28	300	500	2500	70.47	*Staph. aureus*
M	82	26.3	3	300	500	1600	66.4	*Staph. aureus*
M	67	27.2	29	300	500	3500	86.12	*Staph. aureus*
M	81	13.7	7	300	500	400	46.45	*Staph. epidermidis*
M	78	23.9	3	300	500	600	65.39	*Staph. aureus*
M	51	37.9	0.75	300	500	600	44.23	*E. faecalis*
F	75	36.0	75	250	500	3200	75.14	*Staph. epidermidis*
M	79	20.7	202	150	500	-1500	65.35	*Staph. aureus*
F	89	25.6	28	300	500	1300	99.25	*Staph. aureus*
M	70	33.1	51	250	250	320	23.5	*E. faecalis*
M	66	16.2	38	150	500	-1500	40.5	*Klebsiella pneumoniae*
M	78	28.0	20	200	500	1000	72.25	*Staph. aureus*
3/12	74.0 [68.0–78.5]	26.3 [24.0–33.2]	28.0 [11.0–45.0]	285 [225–300]	500 [500–500]	600 [160–1450]	66.4 [45.7–80.6]	

### Primary outcome

**Fig. 1:**
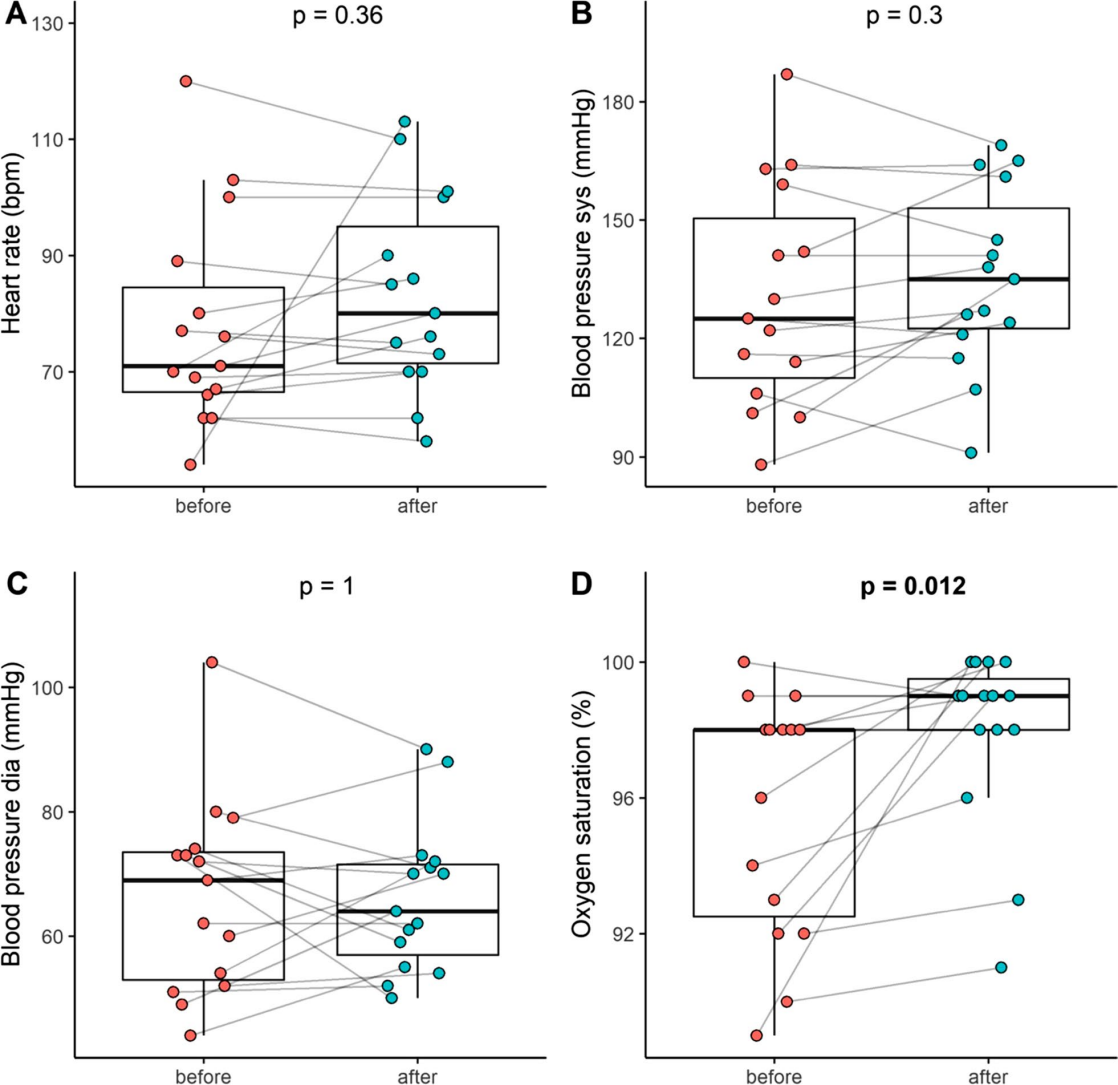
Vital signs including heart rate (**A**), systolic blood pressure (**B**), diastolic blood pressure (**C**) and oxygen saturation (**D**) before and after treatment with the Seraph® 100

**Fig. 2:**
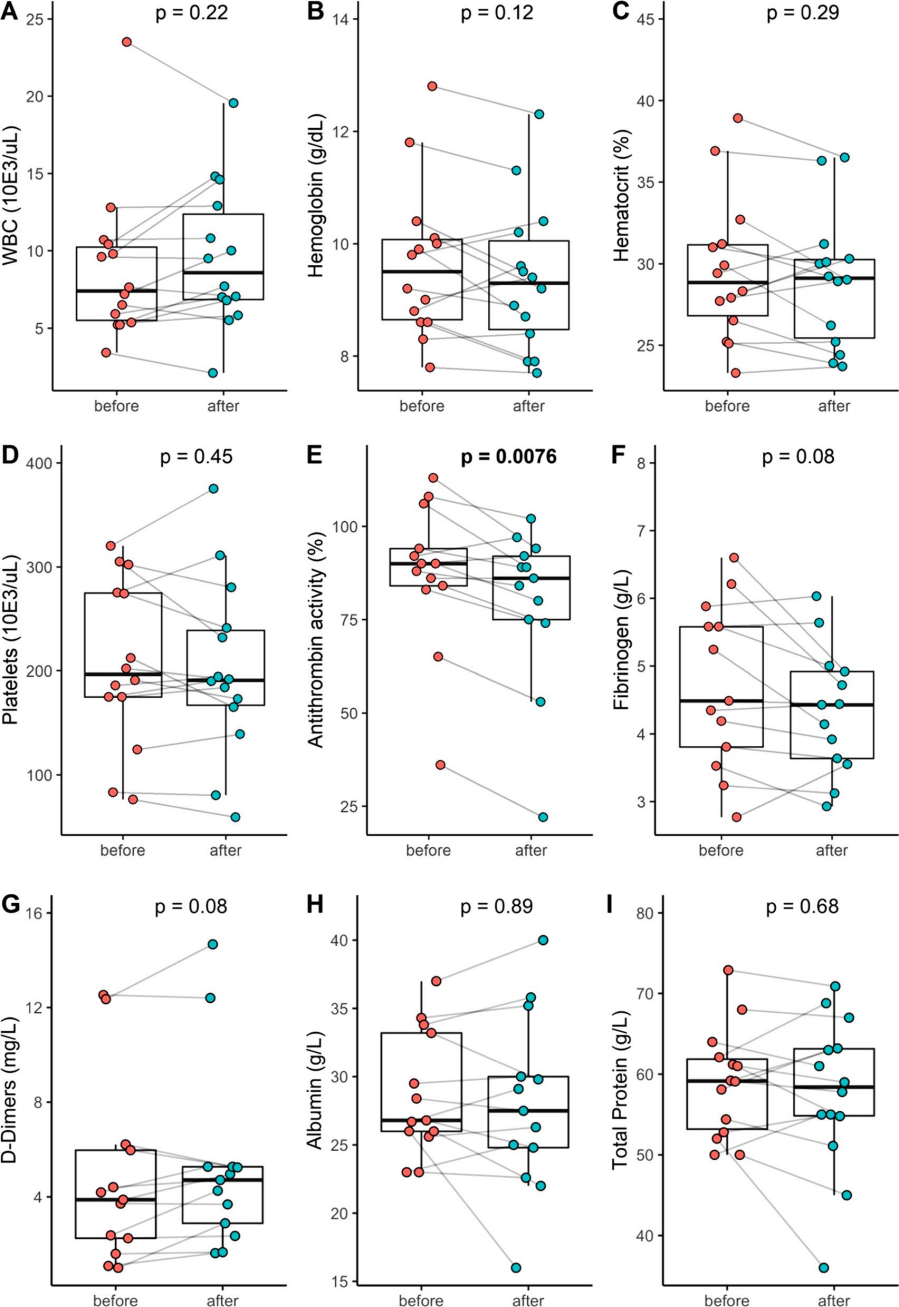

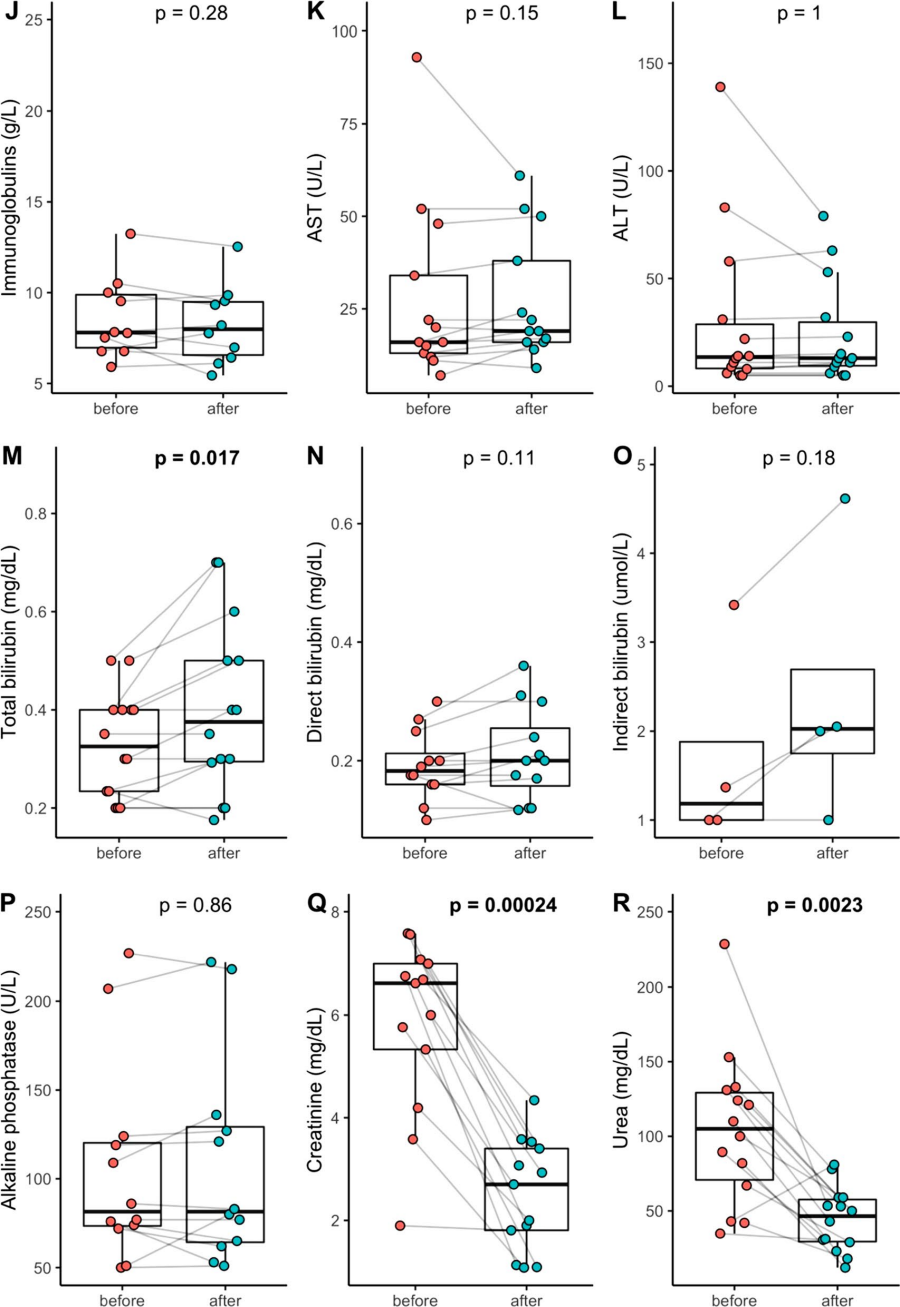
Routine blood tests of white blood cell count (**A**), hemoglobin (**B**), hematocrit (**C**), platelets (**D**), antithrombin activity (**E**), fibrinogen (**F**), D-dimers (**G**), albumin (**H**), total protein (**I**), immunoglobulins (**J**), aspartate transaminase (**K**), alanine transaminase (**L**), total bilirubin (**M**), direct bilirubin (**N**), indirect bilirubin (**O**), alkaline phosphatase (**P**), creatinine (**Q**) and urea (**R**) before and after Seraph® 100 therapy

All patients tolerated the treatment well. None of the procedures had to be terminated prematurely. There were no visible clots or blood remnants reported in the Seraph® 100 after the rinse back with normal saline at the end of treatment. All patients survived the follow up period. A pre- and post- treatment overview of all vital signs and routine serum tests is given in Figs. 1 and 2.

During the treatment with the Seraph® 100 we did not observe any adverse effect on vital signs. Blood pressure and heart rate remained stable, peripheral oxygen saturation did increase from a median of 98.0 [92.5–98.0] before the treatment to 99.0 [98.0–99.5] %; *p* = 0.0184 (Fig. 1D). Antithrombin activity declined from 90.0 [84.5–103.0] to 85.0 [74.25–91.25] %; *p* = 0.0076 during the combined dialysis and Seraph® 100 treatment (Fig. 2E). As expected in a hemodialysis session, we detected a decrease for serum creatinine from 6.31 [4.54–6.94] mg/dl to 2.82 [1.83–3.46] mg/dl; *p* < 0.0017 and for urea from 100.0 [74.5–127.5] mg/dl to 46.5 [29.4–57.6] mg/dl; *p* = 0.0023 (Fig. 2Q and R), Serum total bilirubin levels increased during the procedure from 0.35 [0.20–0.40] mg/dl to 0.4 [0.28–0.52] mg/dl; *p* = 0.0168) (Fig. 2M). A single adverse event was reported during the procedures, but was not related to the device or the procedure according to the reporting clinician. Reported adverse events (AE) during the 14 day observation period are listed in Additional file 1: Table S2. No adverse events AE was supposedly related to the procedure.

### Secondary outcome

**Fig. 3:**
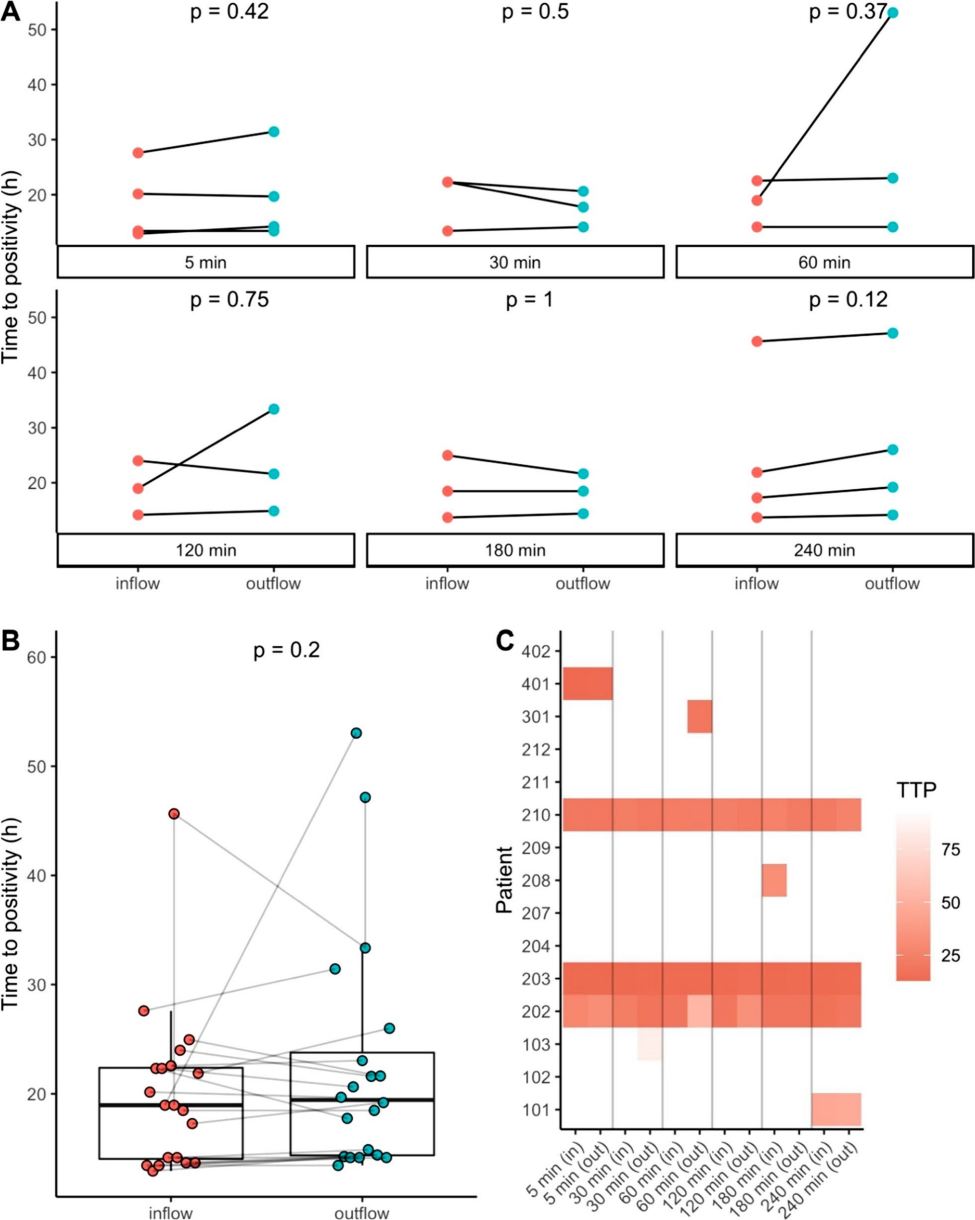
Overview of the change in time to positivity (TTP) during the treatment. **A** shows inflow and outflow TTP values of the Seraph® 100 at every time point when positive blood cultures at inflow and outflow were measured. **B** shows all positive inflow and outflow blood cultures with their respected TTP. **C** shows a heat map of the course of the TTP in the inflow (in) and outflow (out) blood cultures at every time point. Red represents a high TTP, white are negative blood cultures

The secondary outcome measure was the reduction of bacteria in blood passed through the Seraph® 100 over the four-hour treatment. We show inflow and outflow TTP values in Fig. 3. A total of four out of fifteen patients (26.7%) still showed bacteremia at the beginning of the treatment, in one patient a positive inflow and outflow blood culture pair could be measured during the procedure after initially showing negative blood cultures. Over the Seraph® 100 treatment, a total of 52 (54.2%) blood cultures showed bacterial growth with a measurable TTP, resulting in 20 inflow and outflow pairs for a paired analysis. Overall, the blood cultures showed a median TTP of 18.96 [14.04–22.38] hours before the device and 19.44 [14.36–23.78] hours after the Seraph® 100 (*p* = 0.2) (Fig. 3B). The median TTP increase between positive inflow and outflow blood cultures was 36 [− 7.2 to 96.3] minutes.

One patient with *Klebsiella pneumoniae* infection who still had positive blood cultures 30 min into the Seraph® 100 treatment turned to be blood culture negative in the subsequent sampling points. A patient by patient overview of the inflow and outflow TTP in the blood culture positive patients is given in Additional file 1: Figure S1.

## Discussion

Our first in human study could show that an extracorporeal biomimetic device can safely and easily be used in conjunction with standard dialysis equipment. Even in acutely infected patients the treatment does not lead to cardiovascular instability. Just a few patients showed positive blood cultures at the beginning of the treatment, so significant pathogen reduction based on changes in TTP could not be proven in this study. However, the secondary efficacy endpoint (median TTP increase of > 22 min) was met.

Currently, the only FDA approved therapy for targeting pathogens in sepsis are anti-infective drugs, but slow pathogen identification and therefore improper empirical use of antibiotics can lead to poor outcome and antibiotic resistance. Extracorporeal treatment approaches in bacteremia or sepsis have failed thus far to improve clinically relevant patient centered outcomes. However, those treatment strategies have so far concentrated at later stages of sepsis and were mainly aimed to modify mediators that were increased in response to a fulminant infection, where treatment success is difficult to achieve [19, 20]. Seraph® 100 is not designed for the late stage of sepsis but for early usage in serious infections that can trigger sepsis. However, strategies for an early implementation of the Seraph® 100 therapy, without the delay of waiting for a positive blood culture, have to be established.

### Safety-vital signs

Even during routine outpatient dialysis procedures, hypotension occurs in almost 50% of the treatments [21]. On this background it is remarkable that the additional placement of the Seraph® 100 in series, upstream of the dialyzer, did not result in drop in blood pressure. An additional finding from the study was a significant increase in peripheral oxygen saturation during the treatment with Seraph® 100 treatment. This data point was not included in the clinical research file but was recorded during the normal hemodialysis procedure. Although we do not provide arterial blood gas analysis, we consider this finding as significant, as the peripheral O_2_ saturation typically decreases during hemodialysis [22, 23].

### Safety-laboratory data

With regard to the clinical laboratory changes over the four hour treatment we did not see any negative safety signal. Creatinine and urea showed the typical drop of small molecules during hemodialysis [24]. The significant increase in bilirubin levels within the normal range is not clear, we did not see significant signs of hemolysis during the treatment. We do not consider this as a point of concern, as higher bilirubin levels are associated with beneficial effects in hemodialysis patients, [25]. Direct bilirubin and liver function were not affected by the Seraph® 100 treatment. None of the three blood cell lines were significantly altered by the Seraph® 100 treatment. The same holds true for fibrinogen, albumin and immunoglobulins, not to mention the electrolytes. The finding that the antithrombin activity is slightly but significantly decreasing during the treatment seems not of clinical significance. All patients showed high D-Dimer levels, non-significantly increasing during Seraph® 100 treatment. To evaluate the possible activation of disseminated intravascular coagulation (DIC), we calculated the DIC-score [26] before and after the treatment. There was no significant increase in the DIC-score during the Seraph® 100 treatment (before: 3 [2–4], after: 3 [3, 4], *p* = 0.17). Although we did not measure drug levels of anti-infectives during this study, neither extensive in vitro studies using human plasma and a Seraph® 100 in a hemoperfusion setting [27, 28] nor anecdotal clinical reports [29] suggest clinical significant removal of anti-infective drugs. Interestingly, this holds true for tacrolimus and mycofenolatemofetil [30]. However, future clinical studies should evaluate a possible drug adsorption by the device in the clinical use.

### Reduction of bacterial load

The Seraph® 100 is aimed to rapidly remove heparin-binding pathogens from circulating blood. Indeed, in-vitro studies have demonstrated that many bacteria, including all of those present in our patients are bound to the Seraph® 100 adsorption media as blood passes over it, allowing the concentration of bacteria to be reduced by up to 85 percent in a single pass of contaminated blood [17]. As the design of this study required a confirmation of a bloodstream infection by a positive blood culture before patient enrolment, up to 138 h passed until the Seraph® 100 treatment was started. However, four out of 15 patients (26.7%) continued to have positive blood cultures despite appropriate antibiotic therapy. In one patient, blood cultures identified a pathogen at the beginning of the treatment and were negative after the Seraph® 100 treatment. The secondary efficacy endpoint (TTP increase of > 22 min) was met in our study, but the TTP increase of pre to post adsorber blood cultures did not reach statistical significance. Although, in-vitro studies showed a 66% pathogen reduction per pass for S*taphylococcus aureus*, a more conservative endpoint (> 40% reduction with an increase in TTP by at least 22 min) was chosen as in-vitro results with a defined spiked pathogen load may not be achievable in the clinical setting. Additionally, a four hour dialysis session with a 40% pathogen removal would result in a > 99% pathogen elimination, in case no further bacteria are released into the blood during the treatment, which seems meaningful. However, TTP reduction of the Seraph® 100 was ultimately not significant with the limited number of positive blood culture pairs in this study.

### Study limitations

We want to point out several limitations of this study. This study was designed to investigate primarily the safety and performance of the Seraph® 100. Due to the delay between initial diagnosis of bacteremia and the start of the adsorber treatment less than half of the measured blood cultures showed positive results and only five of the treated patients could be included into our efficacy analysis. Therefore, our results regarding the pathogen reduction of the device are limited. All patients in this study were treated by intermittent hemodialysis in conjuction with the Seraph® 100 treatment and all patients received heparin for anticoagulation. Therefore, other renal replacement therapy modalities could not be investigated in this study. However, outside of this investigation the device has been used in the setting of prolonged intermittent renal replacement therapy [29], continues kidney replacement therapy [31] and standalone hemoperfusion [32].

### Outlook

Future studies are needed to assess the efficacy of the device. Such studies should aim to reduce the time between clinical diagnosis of a blood stream infection and the Seraph® 100 treatment. Shortening the time between diagnosis and start of treatment seems to be crucial, as it is for any therapy against bacteria. Another interesting use could be the treatment of prolonged bacteremia as in endocarditis or MDR pathogens, which was no perquisite for this study. Larger clinical trials assessing clinical relevant end-points like incidence of endocarditis, sepsis, hospital length of stay and mortality are currently underway.

## Conclusions

This is the first in human study of hemodialysis patients with proven bacteremia treated with the Seraph® 100. Serial use of the device on a non-modified dialysis machine was technically easy to implement and well tolerated. Although the efficacy endpoint for pathogen reduction was met, further studies are needed to evaluate the clinical efficacy of the device.

## Supplementary Information

**Additional file 1: Table S1** provides detailed inclusion and exclusion criteria of the study. **Table S2** provides detailed information on every reported adverse event in the study. **Figure S1** shows the inflow and outflow TTP of the Seraph® 100 in every patient who started with positive blood cultures.

## Data Availability

The data that support the findings of this study are available from ExThera Medical but restrictions apply to the availability of these data, which were used under license for the current study, and so are not publicly available. Data are however available from the authors upon reasonable request and with permission of ExThera Medical.

## Abbreviations

AE: Adverse event
CFU: Colonizing blood units
DIC: Disseminated intravascular coagulation
FDA: Federal drug agency
MDR: Multi drug resistance
SAE: Severe adverse event
TTP: Time to positivity
