# Supplementary material for: Safety and efficacy of the Seraph® 100 Microbind® Affinity Blood Filter to remove bacteria from the blood stream: results of the first in human study
Source: Crit Care. 2022 Jun 17;26:181. doi: 10.1186/s13054-022-04044-7 (PMC9205040; doi:10.1186/s13054-022-04044-7)

Supplement

## Supplemental table 1: List of inclusion and exclusion criteria

| Inclusion criteria |
| --- |
| 1. Require renal replacement therapy. |
| 2. Be ≥ 18 years old and ≤ 90 years old |
| 3. Positive blood culture and one of the following:  a. Clinical evidence of a catheter exit site to tunnel infection as evidenced by redness, tenderness or purulence.  b. Bacteremia is proven with two separate blood cultures from independent vein punctures.  c. A blood culture where the time to positivity is within 14 hours.  d. Growth from a blood culture taken from the hemodialysis catheter 2 or more hours before the growth of a blood culture drawn peripherally at the same time.  NOTE: Written results for bacteremia and pathogen identification do not necessarily need to come from the hospital lab and written results can be provided by an outside clinic. The test results from the external source should have been generated within one (1) week prior to hospital admission. |
| 4. The patient or patient’s legal representative is able to understand the requirements of the study and signs an approved informed consent form prior to enrollment which explains aggressive care. |
| Exclusion criteria |
| 1. Have an arteriovenous polytetrafluoroethylene (PTFE) graft. |
| 2. Lack of a commitment to full aggressive support. |
| 3. Have inability to maintain a minimum mean arterial pressure of ≥ 65 mm Hg despite vasopressor therapy and fluid resuscitation. |
| 4. Have had chest compressions as part of cardiopulmonary resuscitation (CPR) |
| 5. Have had an acute myocardial infarction (MI) within the past 3 months. |
| 6. Have had serious injury within 36 hours of screening. |
| 7. Have uncontrolled hemorrhage. |
| 8. Are not expected to live > 14 days. |
| 9. Have malignancy and are not expected to live 42 days. |
| 10. Have neutropenia (absolute neutrophil count <500 cells/μL). |
| 11. Have Child-Pugh Class C cirrhosis. |
| 12. Have New York Heart Association Class IV Heart Failure or an ejection fraction <30%. |
| 13. Have known Antithrombin III deficiency. |
| 14. Have platelet count <30,000/μL |
| 15. Cannot have intravenous (IV) supplemental iron halted during trial period. |
| 16. Are currently involved in an investigational drug or device trial. |
| 17. Have been previously enrolled in this clinical trial. |
| 18. Next hemodialysis treatment will not take place for at least 24 hours after enrollment. |
| 19. Serious bleedings and clotting disorders, determined by blood transfusion of > 2 units of packed red blood cells, or, An acute (48 h) hemoglobin decline of at least 2 g/dL, transfusion requirement of >4 units over 48 h, objective evidence of bleed, documented by physician. |
| 20. Breast feeding and pregnant women |
| 21. Contraindications for heparin sodium for injection are:  a. Have heparin sensitivity  b. Severe thrombocytopenia.  c. With an uncontrolled active bleeding state, except when this is due to disseminated intravascular coagulation.  d. In whom suitable blood coagulation tests, e.g. whole blood clotting time, partial thromboplastin time, etc cannot be performed at appropriate intervals (this contraindication refers to full-dose heparin; there is usually no need to monitor coagulation parameters in patients receiving low-dose heparin) |
| 22. Serious injuries, which have occurred more than 36 hours, have to be excluded. |

## Supplemental table 2: List of individual adverse events and their degree as well as their relationship to the procedure.

| Adverse event | SAE | AE during procedure | Related to device | Severity of event | Related to procedure |
| --- | --- | --- | --- | --- | --- |
| AENAMIA | No | No | No | MILD | No |
| ANAEMIA | No | No | No | MILD | No |
| ANAEMIA | No | No | No | MILD | No |
| BACKACHE | No | No | No | MILD | No |
| BLEEDING POSTOPERATIVE | No | No | No | MILD | No |
| EDEMA | No | No | No | MILD | No |
| ENDOCARDITIS | No | No | No | MODERATE | No |
| HEMATOMA | No | No | No | MILD | No |
| HYPERKALEMIA | No | No | No | MILD | No |
| HYPERKALEMIA | No | No | No | MILD | No |
| HYPERPHOSPHATEMIA | No | No | No | MILD | No |
| HYPERTENSION | No | No | No | MILD | No |
| INCREASE OF SIZE OF VALVULAR VEGETATION | No | No | No | MILD | No |
| LOW BLOOD FLOW DIALYSIS | No | Yes | No | MILD | No |
| LUMBAR BODY FRACTURE | Yes | No | No | MODERATE | No |
| EDEMA | No | No | No | MILD | No |
| PLEURA EMPYEMA | Yes | No | No | MODERATE | No |
| PROGRESSIVE ANEMIA | No | No | No | MILD | No |
| PRURITUS | No | No | No | MILD | No |
| RALES | No | No | No | MILD | No |
| SURGICAL REVISION OF AVFISTULA | Yes | No | No | MILD | No |
| SYSTOLIKUM | No | No | No | MILD | No |
| SYSTOLIKUM | No | No | No | MILD | No |
| TACHYARRHYTHMIA ABSOLUTA | No | No | No | MILD | No |
| THROMBOSIS V. JUGULARIS | Yes | No | No | MILD | No |
| UNSUCCESSFUL ATRIAL LINE IMPL. | Yes | No | No | MODERATE | No |

## Supplemental figure 1: TTP at the inflow and outflow site of the Seraph^®^ 100 in all patients who started with positive blood cultures.


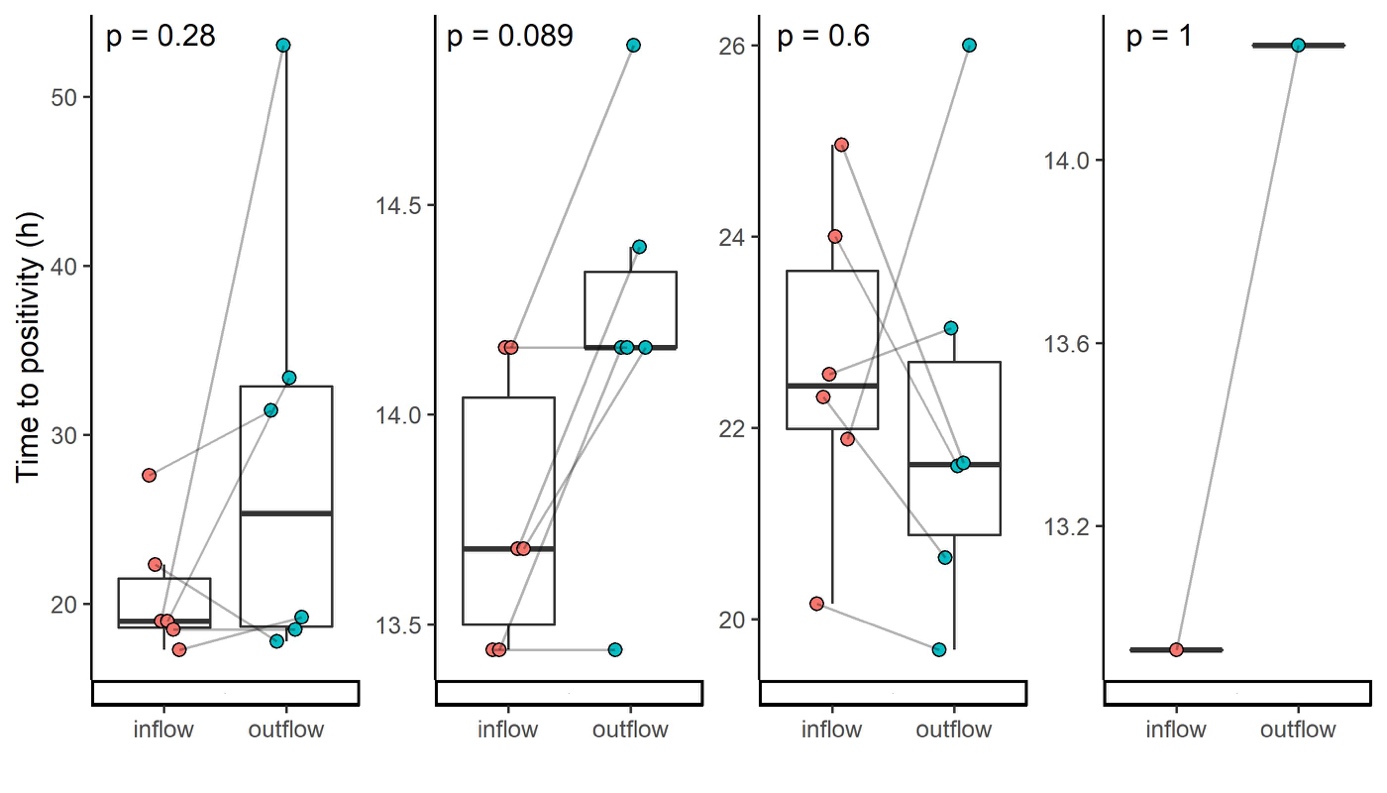

Supplement: Supplementary file 1 — Additional file 1: Table S1 provides detailed inclusion and exclusion criteria of the study. Table S2 provides detailed information on every reported adverse event in the study. Figure S1 shows the inflow and outflow TTP of the Seraph® 100 in every patient who started with positive blood cultures. [file 13054_2022_4044_MOESM1_ESM.docx]
